# Evaluation of interleukin-23 receptor (IL-23R) gene polymorphisms and serum IL-23 levels in patients with psoriasis

**DOI:** 10.3906/sag-1904-48

**Published:** 2019-10-24

**Authors:** Başak FİLİZ, Mehmet YILDIRIM, Kuyaş HEKİMLER ÖZTÜRK, Fevziye Burcu ŞİRİN, Seda ÇELİK, İjlal ERTURAN, Selma KORKMAZ, Hikmet ORHAN

**Affiliations:** 1 Department of Dermatology, Faculty of Medicine, Süleyman Demirel University, Isparta Turkey; 2 Department of Medical Genetics, Faculty of Medicine, Süleyman Demirel University, Isparta Turkey; 3 Department of Biochemistry, Faculty of Medicine, Süleyman Demirel University, Isparta Turkey; 4 Department of Biostatistics and Medical Informatics, Faculty of Medicine, Süleyman Demirel University Isparta Turkey

**Keywords:** Psoriasis, serum IL-23 levels, IL-23R gene polymorphisms

## Abstract

**Background/aim:**

IL-23R gene polymorphisms and the association of these polymorphisms with serum IL-23 levels were investigated in patients with psoriasis in the current study.

**Materials and methods:**

Sixty-seven patients with psoriasis who were admitted to our dermatology outpatient clinic and 67 healthy controls were included in the study. Polymorphisms of the IL-23R gene were determined by KASP-PCR method, and serum IL-23 levels were determined by ELISA method.

**Results:**

The distribution of IL-23R gene polymorphisms rs2201841, rs11209026, rs7530511, rs1343152, and rs11465804 was not significantly different in the patient and control groups. The AA genotype of the rs2201841 locus in males and the GA genotype in females, as well as the AA genotype of the rs1343152 locus in males and the CA genotype in females, were statistically significant in patients with psoriasis. The mean serum IL-23 level was significantly lower in the patient group (42.62 ± 5.96) compared to the control groups (75.76 ± 13.24).

**Conclusion:**

IL-23R gene polymorphisms including rs2201841, rs11209026, rs7530511, rs11465804, and rs1343152 were not found to be significantly related to psoriasis. Different genetic polymorphisms may play a role in the development of psoriasis in female and male populations. Ethnic differences between different populations may have led to differences in the distribution of polymorphisms in the current study with compared to other published studies. Additionally, many different genes, polymorphisms, and environmental factors that have an effect on the development of psoriasis may affect the disease process.

## 1. Introduction

Psoriasis is a chronic inflammatory skin disease characterized by papules and/or plaques that are often covered with silvery-whitish scales [1]. The presence of a family history of psoriasis in about 30% of patients, simultaneous presence of psoriasis in monozygotic twins, and association of early-onset psoriasis with many major tissue antigen alleles, especially human leukocyte antigen (HLA) Cw6, are some of the factors that suggest a role for genetic factors in the occurrence of the disease [2,3].

Although the pathogenesis of psoriasis is not known exactly, dysregulation in T cells is thought to induce keratinocyte proliferation. Studies have shown a prominent role of the helper T-cell (Th) 17/interleukin (IL)-23 pathway in the pathogenesis of psoriasis [4,5]. IL-23 is the key cytokine that directs naive CD4+ T cells to differentiate into Th17 cells by upregulating the IL-23 receptor (IL-23R) on naive T cells, resulting in the secretion of cytokines [4,5]. 

Previous studies have shown a significant relationship between psoriasis and some polymorphisms in the IL-23R gene [6,7]. The IL-23R gene consists of 11 exons and 10 introns; it is localized in chromosome 1p31.3 and is 2912 bp long (Figure). The rs2201841, rs11209026, rs11465804, rs7530511, and rs1343152 polymorphisms are located in the 7th intron, 9th exon, 8th intron, 7th exon, and 8th intron, respectively.

Our study is the first to investigate the relationship between IL-23R gene polymorphisms and psoriasis in Turkey. Additionally, our study is the first in the literature to investigate the relationship between polymorphisms rs11465804 and rs1343152 and psoriasis, and also the first to investigate the relationship between serum IL-23 levels and rs1343152 rs11209026, rs7530511, and rs11465804 polymorphisms in patients with psoriasis.

The aim of this study was to investigate IL-23R gene polymorphisms (rs2201841, rs11209026, rs7530511, rs11465804, rs1343152) and the relationship of these polymorphisms with serum IL-23 levels and clinical features of Turkish patients with psoriasis (age at onset, duration and severity of disease, family history, and nail and joint involvement). 

## 2. Materials and methods

### 2.1. Study subjects

The study was initiated after approval from the Süleyman Demirel University Medical Faculty Clinical Research and Ethics Committee (decision date 27.12.17 and number 187). An informed consent form was signed by all subjects of both the patient and control groups. The study included 67 patients aged ≥18 years who were admitted to the dermatology clinic between December 2017 and August 2018 and 67 healthy controls aged ≥18. Patients with a clinical and/or histopathological diagnosis of psoriasis, who did not undergo any systemic treatment in the last 3 months or topical treatment for the last 1 month, and who did not have any inflammatory disease other than psoriasis were included in this study. Individuals who did not have any known disease and did not use any medication were included in the control group. Cigarette and alcohol users, breastfeeding and pregnant women, and individuals who were unwilling to participate were excluded from the study.

### 2.2. Sample collection and preparation

Peripheral venous blood samples from the patient and control groups were collected into two blood collection tubes (EDTA-containing and gel-included biochemistry tubes) after a fasting period of at least 8 h.

Gel-included biochemistry tubes were centrifuged at 3000 rpm for 10 min and the serum was collected from these samples. The serum samples were stored at –80 °C until the determination of serum IL-23 levels by an ELISA kit in the Medical Biochemistry Laboratory.

Genomic DNA was isolated from whole blood samples using a commercially available DNA extraction kit (RTA, DNA and RNA Isolation Kit) according to the manufacturer’s instructions. Isolated genomic DNA was analyzed by 0.8% agarose gel electrophoresis to evaluate the DNA quality; DNA quantity was assessed using a NanoDrop Spectrophotometer (Thermo Scientific, Waltham, MA, USA). The DNA samples were stored at –80 °C until the determination of IL-23R gene single nucleotide polymorphisms (rs2201841, rs11209026, rs7530511, rs11465804, rs1343152) by polymerase chain reaction (PCR) in the Medical Genetics Laboratory.

### 2.3. Measurement of serum IL-23 levels

Quantitative measurement of serum IL-23 levels was performed using a commercial ELISA kit according to the manufacturer’s instructions (Shanghai Sunred Biological Technology Co., Ltd., Shanghai, China). A calibration curve was generated by measuring the standard concentrations and the IL-23 concentration in each sample was determined by interpolation from the calibration curve. The detection limit of the assay was 0.522 pg/mL.

### 2.4. DNA genotyping

Analysis of IL-23R gene polymorphisms rs2201841, rs11209026, rs7530511, rs11465804, and rs1343152 was performed by competitive allele-specific PCR (KASP) assay. All the primers were synthesized by LGC Genomics (Hoddesdon, UK). Primer sequences are given in Table 1. Reactions were performed according to the following standard KASP PCR program: activation at 94 °C for 15 min, then 10 touchdown cycles of 94 °C for 20 s (denaturing), 61–55 °C for 60 s (annealing and elongation), and 23 °C for 30 s (plate reading), followed by 26 cycles of 94 °C for 20 s, 55 °C for 60 s, and 23 °C for 30 s. Fluorescence was tracked in real time with plates read at the end of every amplification cycle. Results were visualized with SNP Viewer software (version 1.99, Hoddesdon, UK). 

**Table 1:** Polymerase chain reaction primers of selected single nucleotide polymorphisms (SNPs) with FAM and HEXfluorescentdyes.

Allele ID	Primer allele(FAM)	Primer allele(HEX)	Allele(FAM)	Allele(HEX)
rs2201841	GTAATAGGAAACTAATATAGAAGATGATGACT	AATAGGAAACTAATATAGAAGATGATGACC	A	G
rs11209026	TTGATTGGGATATTTAACAGATCATTCCA	GGGATATTTAACAGATCATTCCG	A	G
rs7530511	GGCAGCCTTGGAGTTCACC	ACTGGCAGCCTTGGAGTTCACT	C	T
rs11465804	AGTATGATGGGTTAAAATGGGCAATTC	CAGTATGATGGGTTAAAATGGGCAATTA	G	T
rs1343152	GATAAGAGGCAGAGTTTAATTCAA	CTGATAAGAGGCAGAGTTTAATTCAC	A	C

### 2.5. Statistical analysis 

Genotype distribution within the groups of patients and controls were compared with values predicted by Hardy–Weinberg equilibrium (HWE) using the chi-square test. HWE was calculated with an online program (http://www.oege.org/software/hwe-mr-calc.shtml). Explanations for HWE are also given on that website. Continuous variables were expressed as mean±SD, and the significance level was defined as P<0.05. Tests for normality were conducted with Kolmogorov–Smirnov test. The Mann–Whitney U test was used for comparing independent groups for demographic and clinical data. The difference of allelic and genotypic frequencies between patient and control groups was estimated by Pearson’s chi-square test and Fisher’s exact test. SPSS 18.0 (SPSS Inc., Chicago, IL, USA) was used to carry out all statistical analyses.

## 3. Results

The patient and control groups had similar age and sex distributions (Table 2). The serum IL-23 levels were significantly lower in the patient group (42.62 ± 5.96) compared to the controls (75.76 ± 13.24) (Table 2). The clinical and laboratory features of individuals are shown in Table 2. There were 44 patients (65.7%) with mild (Psoriasis Area Severity Index (PASI) score of <10), and 23 patients (34.3%) with moderate-severe (PASI ≥10) psoriasis. Fourteen patients (20.9%) had nail changes such as subungual hyperkeratosis, onycholysis, pitting, and oil drop appearance due to psoriasis. One patient had psoriatic arthritis, and 21 (31.3%) patients had a family history of psoriasis (Table 2). Serum IL-23 levels were not significantly correlated with sex, age, duration of disease, PASI score, nail and joint involvement, or family history in the patients or the controls. 

**Table 2:** Descriptive and comparison statistics of the demographic and clinical features of patients and controlsPASI: Psoriasis area severity index, SD: standard deviation, n: number.

	Patients (n = 67)	Controls (n = 67)	P
Male/female, n (%)	37/30 (55.2%)	31/36 (46.3%)	0.149
Age, years, mean ± SD	40.55 ± 14.83	36.00 ± 13.07	0.062
Initial age of disease, mean ± SD	31.72 ± 1.94	-	
Duration of the disease, years, mean ± SD	8.90 ± 0.93	-	
PASI, mean ± SD PASI<10, n (%)PASI ≥10, n (%)	10.49 ± 1.3444 (65.7)23 (34.3)	-	
Serum levels of IL-23, mean ± SD	42.62 ± 5.96	75.76 ± 13.24	0.024
Family history, n (%)	21 (31.3)		
Nail involvement, n (%)	14 (20.8)		
Psoriatic arthritis, n (%)	1 (1.4)		

The distribution of IL-23R gene polymorphisms rs2201841, rs11209026, rs7530511, rs1343152, and rs11465804 was not significantly different in the patient and control groups (Table 3). Polymorphisms rs2201841 and rs1343152 in 14 patients (7 patients, 7 controls), and polymorphisms rs11209026, rs7530511, and rs11465804 in thirteen patients (7 patients, 6 controls) could not be studied due to technical reasons.

**Table 3:** Genotype distributions of rs2201841, rs11209026, rs7530511, rs11465804, and rs1343152.HWE: Hardy–Weinberg equilibrium, n: number.

Genotypes	Patients, n (%)	Controls, n (%)	P
rs2201841			
AA	16 (26.7%)	17 (28.4%)	0.673
GA	29 (48.3%)	32 (53.3%)	
GG	15 (25%)	11 (18.3%)	
HWE	0.797	0.548	0.605
rs11209026			
GG	58 (96.7%)	57 (93.4%)	0.680
GA	2 (3.3%)	4 (6.6%)	
AA	0	0	
HWE	0.895	0.791	0.684
rs7530511			
CC	49 (81.7%)	50 (82%)	0.592
TC	10 (16.7%)	11 (18%)	
TT	1 (1.6%)	0	
HWE	0.566	0.438	0.830
rs11465804			
TT	55 (91.7%)	58 (95.1%)	0.491
TG	5 (8.3%)	3 (4.9%)	
GG	0	0	
HWE	0.736	0.843	0.498
rs1343152			
AA	13 (21.7%)	15 (25%)	0.690
CA	30 (50%)	32 (53.3%)	
CC	17 (28.3%)	13 (21.7%)	
HWE	0.972	0.599	0.519

The rs2201841 AA genotype was found to be statistically significantly higher in males while the GA genotype was statistically significantly higher in female patients with psoriasis (P = 0.021). No such difference in genotype was detected in the control group. The rs1343152 variant AA genotype was found to be statistically significant in males and the CA genotype in female patients with psoriasis (P = 0.027); again, no such difference in genotype was detected in the control group. There was no significant relationship between genotypes of rs11209026, rs7530511, and rs11465804 variants and sex in patients with psoriasis (Table 4). 

**Table 4:** Relationship between genotypes and sex.n: Number.

Alleles	Groups	Genotypes	Female, n (%)	Male, n (%)	P
rs2201841	Patients (n = 60)	AA	3 (18.8)	13 (81.3)	0.021	GA	18 (62.1)	11 (37.9)	GG	7 (46.7)	8 (53.3)	Controls (n = 60)	AA	8 (47.1)	9 (52.9)	0.576	GA	20 (62.5)	12 (37.5)	GG	6 (54.5)	5 (45.5)
		rs11209026	Patients (n = 60)	AA		-	-	0.178	GA	-	2 (100.0)		GG	28 (48.3)	30 (51.7)		Controls (n = 61)	AA	-	-	0.442	GA	3 (75.0)	1 (25.0)	GG	31 (54.4)	26 (45.6)
				rs7530511		Patients (n = 60)	CC		23 (46.9)	26 (53.1)	0.631		TC	5 (50.0)	5 (50.0)			TT	-	1 (100.0)		Controls (n = 61)	CC	26 (52.0)	24 (48.0)	0.210	TC	8 (72.7)	3 (27.3)	TT	-	-
	rs11465804				Patients (n = 60)		TT		26 (47.3)	29 (52.7)		0.755	TG	2 (40.0)	3 (60.0)	GG		-	-	Controls (n = 61)			TT	32 (55.2)	26 (44.8)		0.696	TG	2 (66.7)	1 (33.3)	GG	-	-
			rs1343152				Patients (n = 60)	AA	2 (15.4)	11 (84.6)			0.027	CA	18 (60.0)	12 (40.0)	CC	8 (47.1)	9 (52.9)		Controls (n = 60)		AA	7 (46.7)	8 (53.3)			0.660	CA	19 (59.4)	13 (40.6)	CC	8 (61.5)	5 (38.5)

Serum IL-23 levels were higher in patients with the rs2201841 variant GA genotype than those with the GA genotype in the control group but this relationship was not statistically significant. The serum IL-23 level of one patient with TT genotype in the rs7530511 variant was found to be lower than the mean serum IL-23 levels of other patients with CC and TC genotypes, but this difference was not statistically significant. There was no statistically significant relationship between serum IL-23 levels and rs11209026, rs1343152, and rs11465804 genotype variants in the patient and control groups (Table 5). Also, there was no statistically significant relationship between age or PASI and rs2201841, rs11209026, rs7530511, rs11465804, and rs1343152 genotype variants (Table 5 and Table 6, respectively).

**Table 5:** Relationship between genotypes and age and IL-23 levels.SD: Standard deviation, n: number.

Alleles	Groups	Genotypes	n	Age, mean ± SD	P	IL-23 levels, mean ± SD	P
RS2201841	Patients	AA	16	40.19 ± 13.78	0.948	31.49 ± 35.44	0.184
		GA	29	40.66 ± 16.39		53.57 ± 56.92	
		GG	15	41.93 ± 15.45		28.67 ± 43.10	
	Controls	AA	17	27.41 ± 8.73	0.657	72.25 ± 96.44	0.978
		GA	32	29.41 ± 8.97		75.94 ± 120.47	
		GG	11	30.18 ± 7.49		81.42 ± 106.85	
rs11209026	Patients	GG	58	40.38±15.24	0.201	41.11 ± 49.34	0.772
		GA	2	54.50±10.61		51.55 ± 69.2	
	Controls	GG	57	28.39 ± 8.42	0.138	79.09 ± 112.16	0.407
		GA	4	35.00±10.13		31.81 ± 15.40	
rs7530511	Patients	CC	49	41.18 ± 15.40	0.916	43.10 ± 51.40	0.826
		TC	10	39.70 ± 16.05		35.66 ± 42.32	
		TT	1	36.00		19.28	
	Controls	CC	50	28.82 ± 8.49	0.999	76.72 ± 113.75	0.912
		TC	11	28.82 ± 9.52		72.66 ± 89.10	
rs11465804	Patients	TT	55	40.29 ± 15.61	0.351	41.35 ± 50.15	0.956
		TG	5	47.00 ± 9.67		42.65 ± 44.83	
	Controls	TT	58	28.62 ± 8.53	0.431	78.41 ± 111.29	0.450
		TG	3	32.67 ± 11.02		29.17 ± 17.72	
rs1343152	Patients	AA	13	39.69 ± 15.13	0.733	32.31 ± 35.36	0.391
		CA	30	39.93 ± 15.66		50.27 ± 56.72	
		CC	17	43.35 ± 15.26		32.91 ± 43.96	
	Controls	AA	15	27.20 ± 8.67	0.348	77.00 ± 102.00	0.955
		CA	32	28.66 ± 8.74		76.55 ± 120.24	
		CC	13	31.85 ± 7.97		73.05 ± 99.79	

**Table 6:** Relationship between genotypes and PASI.PASI: Psoriasis Area Severity Index.

Alleles	Genotypes	PASI <10, n (%)	PASI ≥10 n (%)	P
rs2201841	AA	11 (68.8)	5 (31.3)	0.976	GA	19 (65.5)	10 (34.5)	GG	10 (66.7)	5 (33.3)
	rs11209026	AA	-		-	0.309	GA	2 (100.0)	0 (0.0)	GG	38 (65.5)	20 (34.5)
		rs7530511	CC		33 (67.3)		16 (32.7)	0.357	TC	7 (70.0)	3 (30.0)	TT	-	1 (100.0)
rs11465804			TT	35 (63.6)	20 (36.4)		0.099		TG	5 (100.0)	0 (0.0)	GG	-	-
	rs1343152		AA	8 (61.5)	5 (38.5)	0.873			CA	20 (66.7)	10 (33.3)	CC	12 (70.6)	5 (29.4)

The distribution of alleles in the rs2201841, rs11209026, rs7530511, rs11465804, and rs1343152 polymorphisms of the IL-23R gene were not significantly different in the patient and control groups (Table 7). Moreover, IL-23R gene polymorphisms were not significantly correlated with clinical features of the patients in this study. 

**Table 7:** Distribution of alleles (rs2201841, rs11209026, rs7530511, rs11465804, rs1343152) by patient and control groups and test statistics.n: Number.

Alleles	Patients, n (%)	Controls, n (%)	P
rs2201841			
Major allele (A)	61 (48.03)	66 (52.21)	0.518
Minor allele (G)	59 (51.97)	54 (47.79)	
rs11209026			
Major allele (G)	118 (50)	118 (50)	0.420
Minor allele (A)	2 (33.33)	4 (66.67)	
rs7530511			
Major allele (C)	108 (49.2)	111 (50.68)	0.794
Minor allele (T)	12 (52.17)	11 (47.83)	
rs11465804			
Major allele (T)	115 (48.52)	122 (51.48)	0.437
Minor allele (G)	5 (62.50)	3 (37.50)	
rs1343152			
Major allele (A)	56 (47.46)	62 (52.54)	0.439
Minor allele (C)	64 (52.46)	58 (47.54)	

## 4. Discussion

Once the importance of the Th17/IL-23 pathway in the pathogenesis of psoriasis was appreciated, many studies were carried out on polymorphisms of cytokines and receptor gene associated with this pathway [8]. Serum IL-23 levels were found to be significantly lower in patients than in controls in the current study. No significant difference was found between the patient and control groups in IL-23R gene polymorphisms including rs2201841, rs11209026, rs7530511, rs11465804, and rs1343152. Additionally, no significant association was found between these polymorphisms and serum IL-23 levels.

Studies in which IL-23 levels were significantly elevated in the serum and skin of patients with psoriasis have suggested a role of IL-23 in disease pathogenesis [9–11]. Bai et al. found no significant difference in serum IL-23 levels between patients with psoriasis and the control group in their metaanalysis [12]. However, Michalak et al. found lower serum IL-23 levels in patients with psoriasis compared to the control group, but this difference was not statistically significant [13]. 

Corroborating the study by Michalak et al., serum IL-23 levels were significantly lower in psoriasis patients than the control group (P = 0.024) in the current study. Serum IL-23 levels were negatively correlated with the duration of disease but this was not statistically significant. Alobaidi et al., who reported a significant negative correlation between serum IL-23 levels and disease duration, found increased IL-23 levels in early psoriasis lesions and suggested that IL-23 was an early mediator that could be implicated in the formation of psoriatic lesions [11]. Since serum IL-23 levels were significantly lower in patients with psoriasis and a negative correlation (albeit statistically not significant) was detected between serum IL-23 levels and disease duration, it suggests that IL-23 was responsible for the initiation of lesions as a primary triggering mediator and that IL-23 was replaced by other cytokines in the later stages of the disease [11]. In addition, antiinflammatory mechanisms occurring during the regression period of psoriatic lesions may also cause a decrease in serum IL-23 levels. It has been shown that serum IL-23 levels are higher in skin lesions of psoriatic patients, suggesting the migration of IL-23 to the skin from the bloodstream, which may lead to a decrease in serum levels of IL-23 [14,15].

There are many studies evaluating the association between psoriasis and IL-23R rs2201841 polymorphism. In a metaanalysis, the rs2201841 (A>G) G allele and GG genotype were found to be associated with psoriasis [16]. Indhumati et al. reported that the CT and CC genotypes were significantly higher in the psoriasis group at the rs2201841 (T>C) locus [7]. In another study, occurrence of the homozygous minor allele (CC) at the locus of rs2201841 (T>C) was significantly higher in patients with psoriasis than in controls and the risk of disease development in CC genotype carriers was reported to increase by 2.4-fold [17]. In some studies, no significant difference was found between the patient and control groups when AA, GA, and GG genotypes were examined at the rs2201841 locus [18,19]. In the current study, the distribution of AA, GA, and GG genotypes and the distributions of alleles between the patient and control groups were not found to be significant at the rs2201841 locus. Since the polymorphisms at the rs2201841 locus are intronic SNPs, it is possible that the polymorphism may not directly affect receptor function. Alternately, different polymorphisms such as CC or CT at the rs2201841 locus may be associated with psoriasis [7,17]. Ethnic differences may also be responsible for the lack of association of disease with this polymorphism in our study since SNPs may vary in each population.

Another IL-23R gene polymorphism that may be associated with psoriasis is rs11209026. The GG genotype at the rs11209026 locus was reported to increase the risk of psoriasis [20]. The IL-23R gene rs11209026 G minor allele frequency was reported to be significantly higher in psoriatic patients [21]. Boyko et al. found no significant difference in allelic frequencies between the patient and control groups at the rs11209026 locus and a minor allele was reported to have a protective effect against the disease [22]. rs11209026 polymorphism was reported to be associated with psoriasis in Europeans, although this polymorphism was not associated with psoriasis in the Asians [23,24]. The rs11209026 G allele was reported to be a risk factor for psoriasis in some populations [21,23]. A difference in allele frequency of the rs11209026 A allele led us to evaluate this as a risky allele for psoriasis in the current study. The distribution of genotypes and alleles at the rs11209026 locus (GG, GA) was also not significant, and the AA genotype was not found in the patient or the control group. Since the rs11209026 AA polymorphism has not been studied in our country, we were unable to compare our results with published data from the same population. Polymorphisms may vary depending on diseases and races. Thus, while the association of polymorphisms with a disease is not significant in one race, it may be a risk factor for the same disease in a different race.

Boyko et al. reported that the C allele in the IL-23R gene rs7530511 locus was evaluated as risky for psoriasis, while the TT, CT, and CC genotypes were not significantly different between the patient and control groups [22]. In a metaanalysis, the rs7530511 T allele was associated with psoriasis and PsA, and the rs7530511 locus was shown to have protective effects on psoriasis [16]. In the current study, the T allele was considered to be risky for psoriasis because the CC genotype was found to be higher in both patient and control groups when compared to other genotypes (TC, TT). No significant difference was found between the CC, TC, and TT genotypes and alleles at the rs7530511 locus between the patients and controls. The TT genotype was found in one patient whose serum IL-23 level was lower than those of patients with CC and TC genotypes. This result suggests that the TT genotype may be associated with serum IL-23 levels, but there is a need for more patient and control group studies to clarify this issue.

The CA and CC genotypes of the IL-23R gene at the rs1343152 locus were reported to be higher in psoriasis patients than in controls; however, the distribution of these genotypes was not significantly different between the patient and control groups [25]. In our study, no significant difference was found between the patient and control groups in the distribution of genotypes of AA, CA, and CC at the rs1343152 locus and TT, TG, and GG genotypes at the rs11465804 locus. The distribution of alleles at the rs1343152 and rs11465804 loci was not significant. 

In the current study, the rs2201841 AA genotype was found to be statistically significant in males with psoriasis while the GA genotype was found to be statistically significant in women with psoriasis (P = 0.021). Similarly, the rs1343152 AA genotype was found to be statistically significant in men while the CA genotype was significant in women in the psoriasis group (P = 0.027). This suggests that different genetic polymorphisms may play a role in the development of psoriasis in female and male populations. Women with the rs2201841 GA and rs1343152 CA genotypes and men with rs1343152 AA and rs1343152 AA genotypes may be susceptible to psoriasis. However, the sex-dependent associations need to be validated in larger sample sizes as well as in other populations.

To our knowledge, there is only one other study in the published literature investigating serum IL-23 levels in psoriasis patients with the rs2201841 (T>C) polymorphism in the IL-23R gene [7]. In the current study, serum IL-23 levels were found to be significantly higher in patients with the CC genotype compared to those with CT and TT genotypes at the rs2201841 locus in the patient group. Corroborating the current study, serum IL-23 levels were also found to be higher in patients with the GA genotype at the rs2201841 locus in the patient group compared to those with the GA genotype in the control group, but this difference was not statistically significant. The relationship between serum IL-23 level and rs2201841 GA genotype can be evaluated more clearly in further studies involving more patients.

Our study has some limitations. A relatively small number of patients and no evaluation of the association of IL-23R gene polymorphisms (rs2201841, rs11209026, rs7530511, rs11465804, rs1343152) with other cytokines (IL-17 and IL-22) were the main limitations of our study. On the other hand, our study was the first investigating the relationship between rs11465804 and rs1343152 polymorphisms and psoriasis and also examining the relationship between serum IL-23 levels and rs1343152 rs11209026, rs7530511, and rs11465804 polymorphisms in patients with psoriasis in the literature. This study is also the first investigating the relationship between IL-23R gene polymorphisms and psoriasis in Turkey.

In conclusion, different results were obtained when our genotyping data and biochemical parameters were compared with the data currently available in the literature. This result may be due to the fact that studies included varying numbers of patients with different ethnic backgrounds. Moreover, interactions of many different genes, polymorphisms, and environmental factors contribute to the development of a disease process. Further studies are needed to investigate IL-23R polymorphisms in serum and skin lesions together with IL-17 levels and to evaluate more candidate IL-23R gene polymorphisms in larger groups of patients with psoriasis. Studies comparing serum and lesional (skin) IL-23 levels will reveal changes in serum IL-23 levels more clearly.
